# Quantum speed limit for arbitrary initial states

**DOI:** 10.1038/srep04890

**Published:** 2014-05-08

**Authors:** Ying-Jie Zhang, Wei Han, Yun-Jie Xia, Jun-Peng Cao, Heng Fan

**Affiliations:** 1 Beijing National Laboratory for Condensed Matter Physics, Institute of Physics, Chinese Academy of Sciences, Beijing 100190, China; 2 Shandong Provincial Key Laboratory of Laser Polarization and Information Technology, Department of Physics, Qufu Normal University, Qufu 273165, China; 3 Innovative Center of Quantum Matter, Beijing 100190, China

## Abstract

The minimal time a system needs to evolve from an initial state to its one orthogonal state is defined as the quantum speed limit time, which can be used to characterize the maximal speed of evolution of a quantum system. This is a fundamental question of quantum physics. We investigate the generic bound on the minimal evolution time of the open dynamical quantum system. This quantum speed limit time is applicable to both mixed and pure initial states. We then apply this result to the damped Jaynes-Cummings model and the Ohimc-like dephasing model starting from a general time-evolution state. The bound of this time-dependent state at any point in time can be found. For the damped Jaynes-Cummings model, when the system starts from the excited state, the corresponding bound first decreases and then increases in the Markovian dynamics. While in the non-Markovian regime, the speed limit time shows an interesting periodic oscillatory behavior. For the case of Ohimc-like dephasing model, this bound would be gradually trapped to a fixed value. In addition, the roles of the relativistic effects on the speed limit time for the observer in non-inertial frames are discussed.

Quantum mechanics acting as a fundamental law of nature imposes limit to the evolution speed of quantum systems. The utility of these limits is shown in different scenarios, including quantum communication^1^, the identification of precision bounds in quantum metrology^2^, the formulation of computational limits of physical systems^3^, as well as the development of quantum optimal control algorithms^4^. The minimal time a system needs to evolve from an initial state to its one orthogonal state is defined as the quantum speed limit time (QSLT). The study of it has been focused on both closed and open quantum systems. For closed system with unitary evolution, a unified lower bound of QSLT is obtained by Mandelstam-Tamm (MT) type bound and Margolus-Levitin (ML) type bound^5^,^6^,^7^,^8^,^9^,^10^. The extensions of the MT and ML bounds to nonorthogonal states and to driven systems have been investigated in Refs. 11,12,13,14,15. The QSLT for nonunitary evolution of open systems is also studied^16^,^17^,^18^. It is shown that a unified bound of QSLT including both MT and ML types for non-Markovian dynamics can be formulated^18^. However, while this unified bound is applicable for a given driving time for the pure initial states, it is not feasible for mixed initial states. As we all know that decoherence and inaccurate operations are indispensable which may result in mixed initial states.

Here, we shall derive a QSLT for mixed initial states by introducing relative purity as the distance measure, which can characterize successfully the speed of evolution starting from an arbitrary time-evolution state in the generic nonunitary open dynamics. Let us consider the states of a driven system in the damped Jaynes-Cummings model starting from a certain pure state which corresponds to a special case of our result, one may observe that the QSLT is equal to the driving time in the Markovian regime^18^. While by calculating the QSLT starting from the time-evolution state at any point in time which is in general a mixed state, it is interesting to find that the QSLT first begins to decrease from the driving time and then gradually increases to this driving time in the Markovian dynamics. So the speed of evolution in the whole dynamical process exhibits an acceleration first and then deceleration process. Additionally in the case of the non-Markovian regime, the memory effect of the environment leads to a periodical oscillatory behavior of the QSLT. We can also focus on the widely used Ohmic-like reservoir spectra to investigate the QSLT for the time-dependent states of the purely dephasing dynamics process. We demonstrate that the QSLT will be reduced with the starting point in time for Ohmic and sub-Ohmic dephasing model, that is to say, the open system executes a speeded-up dynamics evolution process. While for the super-Ohmic environments, due to the occurrence of coherence trapping^19^, we specifically point out that this QSLT would be gradually trapped to a fixed value, and therefore leads to a uniform evolution speed for the open system. We remark that the findings of those phenomena rely on our general result of QSLT for arbitrary initial states. Finally, we also investigate the influence of the relativistic effect on the QSLT for the observer in non-inertial frames in the above two quantum decoherence models.

## Results

### Quantum speed limit time for mixed initial states

In the following, we shall consider a driven open quantum system and look for the minimal time that is necessary for it evolve from a mixed state *ρ_τ_* to its final state 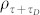. Under the general nonunitary quantum evolutions of open system, also the final state 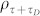 will be generally mixed. One general choice of distance measure between two mixed states *ρ_τ_* and 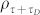 is fidelity 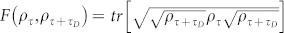. In this case of the initially mixed state *ρ_τ_* should be treated by purification in a sufficiently enlarged Hilbert space. And the fidelity can be written 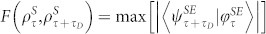, where the maximization is over all pure states 
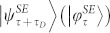
 on a larger Hilbert space that are purifications of the mixed states 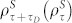 defined in the smaller system *S*. However, performing the optimization over all possible purifications is a challenging task that will be very hard to perform in the general case.

Here we consider the relative purity as a distance measure to derive lower bound on the QSLT for open quantum systems. The so-called relative purity *f*(*τ*) between initial and final states of the quantum system is defined as^20^ 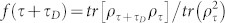. To evaluate the QSLT, let us now characterize the derivative of the relative purity, 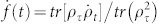. The rate of change of *f*(*t*) will serve as the starting point for our derivation of ML and MT type bounds on the minimal evolution time of an initially mixed state *ρ_τ_*, with the help of the von Neumann trace inequality and the Cauchy-Schwarz inequality, respectively.

By using the von Neumann trace inequality, we begin to provide a derivation of ML type bound to arbitrary time-dependent nonunitary equation of the form 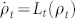. Let us consider such the evolution, 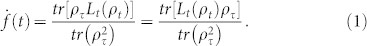
Then, we introduce the von Neumann trace inequality for operators which reads^21^,^22^, 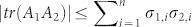, where the above inequality holds for any complex *n* × *n* matrices *A*_1_ and *A*_2_ with descending singular values, *σ*_1,1_ ≥ … ≥ *σ*_1,*n*_ and *σ*_2,1_ ≥ … ≥ *σ*_2,*n*_. The singular values of an operator *A* are defined as the eigenvalues of 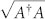
22. In the case of Hermitian operator, they are given by the absolute value of the eigenvalues of *A*. We thus find 
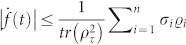
, with *σ_i_* being the singular values of *L_t_*(*ρ_t_*) and 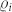 for the initial mixed state *ρ_τ_*. Since the singular values of *ρ_τ_* satisfy 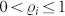, the trace norm of *L_t_*(*ρ_t_*) would satisfy 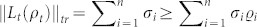, so 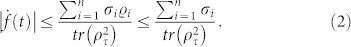
Integrating Eq. (2) over time from *t* = *τ* to *t* = *τ* + *τ_D_*, we arrive at the inequality 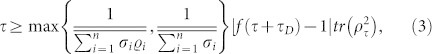
where 
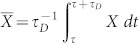
. For unitary processes, 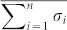 is equal to the time-averaged energy *E*, so the ML bound for closed systems can be expressed 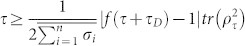.

By noting the following inequality holds 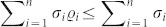, then 
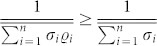
. So we can simplify Eq. (3) as 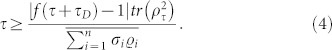
Regarding general nonunitary open system dynamics, Eq. (4) expresses a ML type bound on the speed of quantum evolution valid for mixed initial states.

Next we will derive a unified bound on the QSLT for the open systems. According to Ref. 17, the rate of change of relative purity can be bounded with the help of the Cauchy-Schwarz inequality for operators, 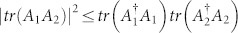. Then 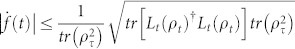, since *ρ_τ_* is a mixed state, 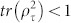, and we obtain 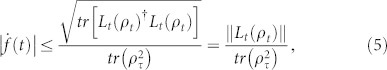
where 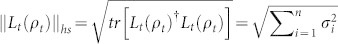 is the Hilbert-Schmidt norm. Integrating Eq. (5) over time leads to the following MT type bound for nonunitary dynamics process, 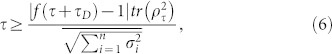
where 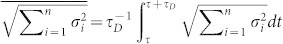 means the time-averaged variance of the energy.

By combining Eqs. (4) and (6), we obtain a unified expression for the QSLT of arbitrary initially mixed states in open systems as follows, 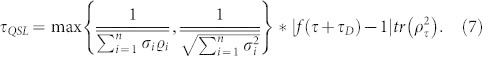
This is one of our main results in this report. For a pure initial state *ρ_τ_*_ = 0_ = |*ϕ*_0_〉〈*ϕ*_0_|, the singular value 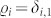, then 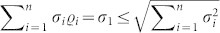. We can find that the expression (7) recovers the unified bound of the QSLT obtained in Ref. 18, which confirms the validity of our results. So *τ_QSL_* formulated in (7) defines the minimal evolution time for arbitrary initial states.

### The speed of evolution in the exactly solvable open system dynamics

In order to clear which bound on the speed limit time *τ_QSL_* can be attained and tight, we must compare 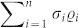 and 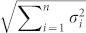. For instance, when 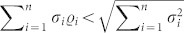, the ML type bound provides the tighter bound on the QSLT. Next we shall illustrate the application of the QSLT to the quantum evolution speed of a qubit system in two decoherence channels. A generally mixed state *ρ*_0_ of a qubit can be written in terms of Pauli matrices, whose coefficients define the so-called Bloch vector 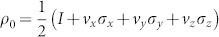, where *I* is the identity operator of the qubit, *σ_k_* (*k* = *x*, *y*, *z*) is the Pauli operator, and 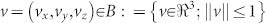.

We firstly consider the exactly solvable damped Jaynes-Cummings model for a two-level system resonantly coupled to a leaky single mode cavity. The environment is supposed to be initially in a vacuum state. The nonunitary generator of the reduced dynamics of the system is 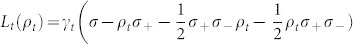, where *σ*_±_ = *σ_x_* ± *iσ_y_* are the Pauli operators and *γ_t_* is the time-dependent decay rate. In the case of only one excitation in the whole qubit-cavity system, the environment can be described by an effective Lorentzian spectral density of the form 
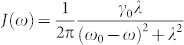
, where *λ* is the width of the distribution, *ω*_0_ denotes the frequency of the two-level system, and *γ*_0_ is the coupling strength. Typically, weak-coupling regime is, *λ* > 2*γ*_0_, where the behavior of the qubit-cavity system is Markovian and irreversible decay occurs. The strong-coupling regime is, *λ* < 2*γ*_0_, where non-Markovian dynamics occurs accompanied by an oscillatory reversible decay. The time-dependent decay rate is then explicitly given by 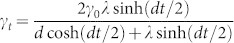, with 
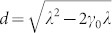
. The reduced density operator of the system at time *t* reads 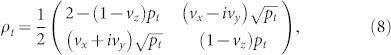
where 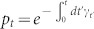.

**Figure 1:**
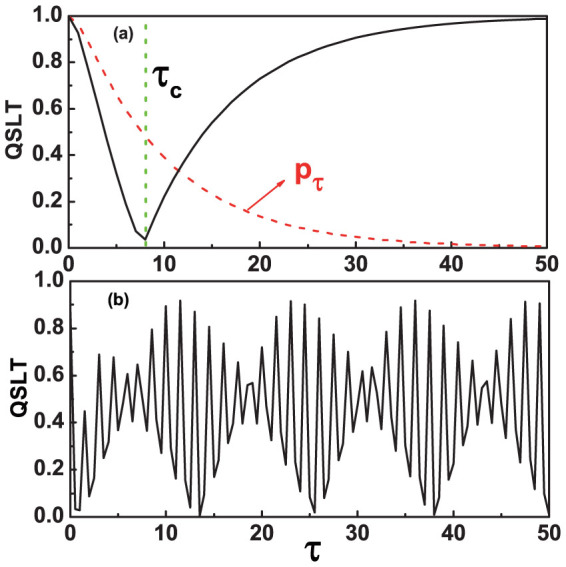
The QSLT for the damped Jaynes-Cummings model as a function of the initial time parameter *τ*. Here we choose the excited state as the initial state, *v_z_* = −1, *v_x_* = *v_y_* = 0. (a) the Markovian regime, *γ*_0_ = 0.1*λ*. (b) the non-Markovian regime, *γ*_0_ = 10*λ*. The red (dashed) line represents the decay of the excited population *p_τ_*. Parameters are chosen as, *ω*_0_ = 1, *λ* = 1 and *τ_D_* = 1.

For the generally mixed state *ρ_τ_* of a qubit, 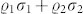 is always less than 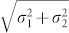, so we reach the result that the ML type bound on the QSLT is tight for the open system. The unified expression (7) proposed for the mixed initial states can demonstrate the speed of the dynamics evolution from an arbitrary time-dependent mixed state *ρ_τ_* to another 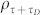 by a driving time *τ_D_*. We examine the whole dynamics process where the system starts from the excited state, *v_z_* = −1 and *v_x_* = *v_y_* = 0. Figs. 1(a) and 1(b) show the QSLT for a time-dependent mixed state *ρ_τ_* as a function of *τ* in the Markovian and non-Markovian dynamics process, respectively, where *τ_D_* = 1. The QSLT can initially decrease to a minimum, and gradually reaches to the driving time *τ_D_* in the Markovian regime. While for the non-Markovian regime, the QSLT decreases to a minimum in the beginning of the evolution, then occurs a periodical oscillatory of the time *τ*. That is to say, in the Markovian regime, the evolution of the qubit first exhibits a speeded-up process for *τ* < *τ_c_* and then shows gradual deceleration process for *τ* > *τ_c_*. In contrast, the speed of evolution for the qubit in the non-Markovian dynamics process complies with an interesting but reasonable periodical oscillatory behavior. This is a newly noticed phenomenon.

The above behavior can be explained by evaluating the QSLT for the qubit to evolve from *ρ_τ_* to 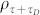, 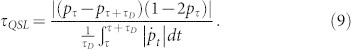
For the Markovian regime *γ_t_* = *γ*_0_, the value of 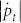 can be given by 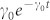, then the speed limit time is simplified as 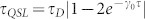. So the appearance seen in Fig. 1(a) depends only on the decay of the excited population 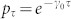 for the time-dependent state *ρ_τ_*, and the critical time 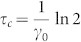. In comparison, the oscillatory behavior shown by Fig. 1(b) in the the non-Markovian regime appears as a consequence of the oscillatory time dependence of the decay rate *γ_t_*.

**Figure 2:**
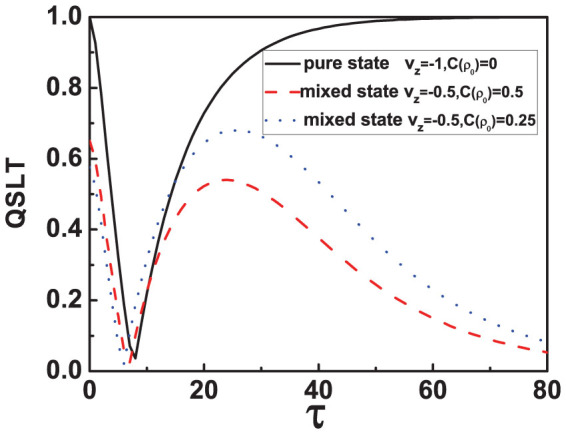
The QSLT for the general mixed states. In the Methods section, we have obtained the QSLT for the qubit to evolve from *ρ_τ_* to 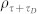 in the damped Jaynes-Cummings model with the specific form 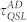. From the expression of 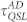, it is easy to show that 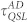 is relate to *v_z_* and the coherence 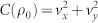 of the initial state. So this figure describe the speed of the qubit dynamics process starting from the general mixed initial states in the damped Jaynes-Cummings model in the Markovian regime (*γ*_0_ = 0.1*λ*). Parameters are *ω*_0_ = 1, *λ* = 1 and *τ_D_* = 1.

As an additional application, when the initial state takes the form of general mixed state, the speed of the qubit dynamics process in the damped Jaynes-Cummings model can also be studied, the results are presented by the dashed red line and dotted blue line in Fig. 2. We can find that in the Markovian regime, the QSLT initially reduces to a minimum and then increases to a maximum. Next, the QSLT would gradually decrease in the following dynamics process. This can be understood that the evolution of the whole dynamics process starting from a mixed initial state exhibits an eventual acceleration process after a speed-up and deceleration process in the Markovian regime.

In what follows, we consider a spin-boson-type Hamiltonian that describes a pure dephasing type of interaction between a qubit and a bosonic environment. It is worth stressing that this qubit-environment model admits an exact solution^23^,^24^. There exists no correlations between the system and the environment at *t* = 0. Furthermore, the environment is initially in its vacuum state at zero temperature. The nonunitary generator of the reduced dynamics of the system is *L_t_*(*ρ_t_*) = *γ_t_*(*σ_z_ρ_t_σ_z_* − *ρ_t_*)/2. By considering that the bosonic environment operator is simply a sum of linear couplings to the coordinates of a continuum of harmonic oscillators described by a spectral function *J*(*ω*)^25^,^26^, then 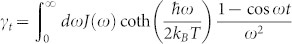. Here, we suppose that the spectral density of the environmental modes is Ohmic-like 
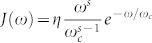
, with *ω_c_* being the cutoff frequency and *η* a dimensionless coupling constant. By changing the *s*-parameter, one goes from sub-Ohmic reservoirs (*s* < 1) to Ohmic (*s* = 1) and super-Ohmic (*s* > 1) reservoirs, respectively. For zero temperature, *t* > 0 and *s* > 0, the dephasing rate can be obtained 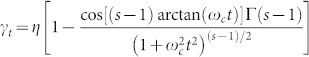, where Γ(*s* − 1) is the Euler Gamma function. Taking the limit *s* → 1 carefully, one also finds 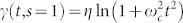. The time evolution of the reduced density matrix of the qubit satisfies 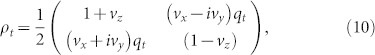
where 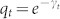.

**Figure 3:**
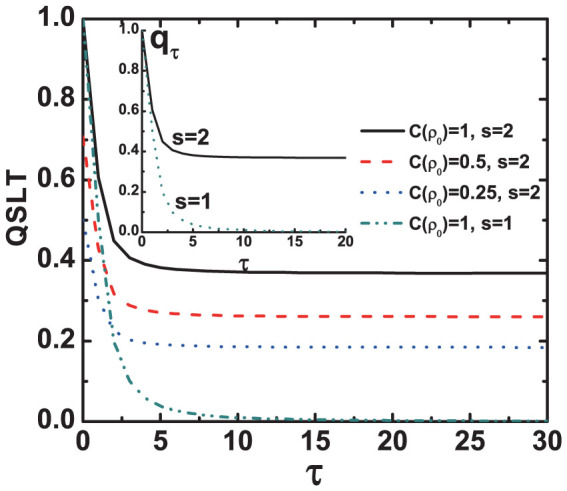
The QSLT for the Ohmic-like dephasing model as a function of the initial time parameter *τ* with different coherence *C*(*ρ*_0_). The inset shows the decay rate *q_τ_* of the coherence for the mixed state *ρ_τ_* with *C*(*ρ*_0_) = 1. Parameters are chosen as, *ω_c_* = 1, *η* = 1 and *τ_D_* = 1.

In this Ohmic-like dephasing model, the QSLT of a qubit can also be described by ML type bound. In the dephasing evolution, by considering an arbitrary mixed state *ρ_τ_* evolves to another 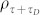 under a driving time *τ_D_*, the QSLT can be calculated 
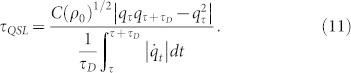
With this, it is easy to show that *τ_QSL_* is independent of *v_z_*, and not only it relates to the dephasing rate of the Ohmic-like environment but also to the coherence of the initial state *ρ*_0_ under a given driving time *τ_D_*. Fig. 3 presents the results of our analysis for *τ_QSL_* in the Ohmic-like dephasing process with different *C*(*ρ*_0_). We observe that, for the same driving time *τ_D_* = 1, the lager coherence of the initial state can decrease the speed of evolution of a quantum system, and thus demand the longer QSLT. By choosing the *s*-parameters which correspond to Markovian regime^27^, the speed limit time can be rewritten as *τ_QSL_* = *τ_D_C*(*ρ*_0_)^1/2^*q_τ_*. Hence, for a given initial state *ρ*_0_, the speed of evolution in the dynamical process is determined by the decay rate *q_τ_* of the coherence for the mixed state *ρ_τ_*. Due to the specific form of the spectral density for Ohmic-like dephasing model^19^, in the case of zero temperature, the qubit dephasing *q_τ_* will predict vanishing coherences in the long time limit for *s* ≤ 1. On the other hand, for *s* > 1 the qubit dephasing *q_τ_* will stop after a finite time, therefore leading to coherence trapping, as shown by the inset of Fig. 3. This is important for quantum information processing since coherence of the quantum state is preserved. Other observations in Fig. 3 are as follows: The open system executes a speeded-up dynamical evolution process in the Ohmic and sub-Ohmic dephasing models. But for the super-Ohmic dephasing model the qubit firstly exhibits a speeded-up dynamical process before a finite time, and then complies with an uniform evolution speed after this finite time.

### Relativistic effect on quantum speed limit time

If the observer for a quantum system in a uniformly accelerated frame with acceleration *a*, the relativistic effect should be taken into account^28^,^29^,^30^,^31^,^32^,^33^,^34^. Here we briefly present the study of the relativistic effect on the QSLT. Owing to the relativistic effect, the coherence of the changing initial state turns into 
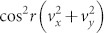
, and becomes much less than that of *C*(*ρ*_0_). So the relativistic effect can increase the speed of evolution of a quantum system in the purely Ohmic-like dephasing channels. The parameter *r* above is defined by 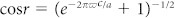, *c* the speed of light in the vacuum, and 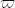 the central frequency of the fermion wave packet. But for the damped Jaynes-Cummings model, in spite of the weaker coherence of the initial state brought by the relativistic effect, the larger excited population 
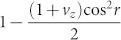
 in the changing initial state can also be acquired. As well as the QSLT mainly depends on the population of excited state under a given driving time in the amplitude-damping channels^35^, the relativistic effect would slow down the quantum evolution of the qubit in the damped Jaynes-Cummings model, therefore leads to a smaller QSLT.

## Discussion

We have derived a QSLT for arbitrary initial states to characterize the speed of evolution for open systems. Some novel phenomena are observed. For the damped Jaynes-Cummings model, we have obtained that the speed of evolution, where the qubit starts from a pure initial state, exhibits an acceleration first and then deceleration process in the Markovian regime, and shows a peculiar periodical oscillatory behavior in non-Markovian regime. Moreover, in the case of the purely dephasing environments, the QSLT would be gradually reduce to a fixed value for the super-Ohmic dephasing model, and hence leads to a uniform evolution speed for the open system. Our results may be of both theoretical and experimental interests in exploring the speed of quantum computation and quantum information processing in the presence of noise.

## Methods

### Quantum speed limit time in open system dynamics

Let us consider the exactly solvable damped Jaynes-Cummings model and the Ohimc-like dephasing model. By considering the initial mixed state 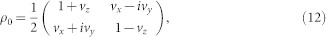
its time evolution reads 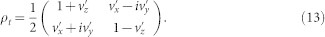
Then we will demonstrate the QSLT for the open system dynamics process from an arbitrary time-dependent mixed state *ρ_τ_* to another 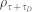 by a driving time *τ_D_*. Due to the unified expression for the QSLT of arbitrary initially mixed states in our work, we first calculate the singular values of *ρ_τ_* and *L_t_*(*ρ_t_*), respectively. For *ρ_τ_*, the singular values 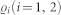 are 
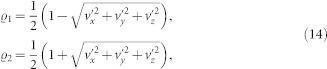
where 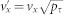, 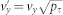 and 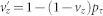 in the damped Jaynes-Cummings model. While for the Ohimc-like dephasing model, 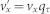, 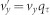 and 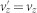. And the singular values *σ_i_* of *L_t_*(*ρ_t_*) can be expressed as 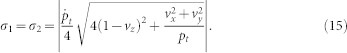
So we can obtain that 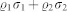 is always less than 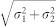, and reach the result that the ML type bound on the QSLT is tight for the open system. The QSLT for the qubit to evolve from *ρ_τ_* to 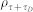 in the damped Jaynes-Cummings model can be obtained 
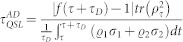
, where 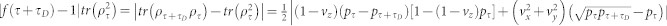. With this expression, it is easy to show that *τ_QSL_* is not only relate to *v_z_* but also to the coherence of the initial state *C*(*ρ*_0_). With regard to the QSLT for the qubit to evolve from *ρ_τ_* to 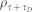 in the Ohimc-like dephasing model, we can obtain as the above derivation 
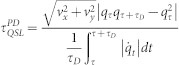
.

### Quantum speed limit time in non-inertial frames

If several detectors or observers are located in different inertial or non-inertial frames, or in curved space-time, the relativistic effect should be taken into account. So in this subsection, we may investigate the influence of the relativistic effect on the QSLT in the damped Jaynes-Cummings model and the Ohimc-like dephasing model. To start with, let us consider the generally mixed state *ρ*_0_ in Eq. (12) of a qubit held by Bob in a non-inertial frame. Now we assume that Bob moves with a uniform acceleration and takes with him a detector for the qubit. From the perspective of Bob, under the single-mode approximation, the Minkowski vacuum state |0〉*_M_* and the Minkowski only excited state |1〉*_M_*, in a uniformly accelerated frame with acceleration *a* are expressed as^30^, |0〉*_M_* = cos*r*|0〉*_I_* |0〉*_II_* + sin*r*|1〉*_I_* |1〉*_II_*, and |1〉*_M_* = |1〉*_I_* |1〉*_II_*. The parameter *r* is defined by 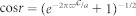, *c* the speed of light in the vacuum, and 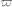 the central frequency of the fermion wave packet. And the subscripts I and II represent the states related to the Rindler-region-I and -region-II respectively. Here *r* ranges from 0 to *π*/4 for 0≤ *a* < ∞. Due to the presence of a Rindler horizon, Bob is forced to trace over a causally disconnected region-II of space-time. Accordingly, the density matrix of the initial state *ρ*_0_ becomes 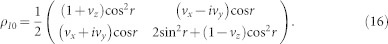
Then the time evolution of the reduced density matrix *ρ_It_* in the damped Jaynes-Cummings model and the Ohimc-like dephasing model can be written as follows, respectively, 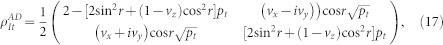
and 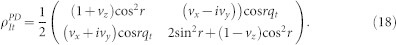
According to the relativistic effect, the QSLT for the qubit to evolve from *ρ*_0_ to *ρ_τ_* in the damped Jaynes-Cummings model can be shown as the form 
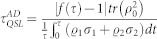
, where 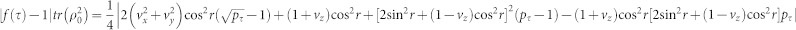, and 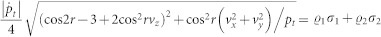. While for the case of the Ohimc-like dephasing model, the QSLT can be calculated 
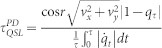
.

## Author Contributions

Y.-J.Z., W.H., Y.-J.X., J.-P.C. and H.F. calculated and analyzed the results. Y.-J.Z. and H.F. co-wrote the paper. All authors reviewed the manuscript and agreed with the submission.

## References

[b1] Bekenstein J. D. Energy cost of information transfer. Phys. Rev. Lett. 46, 623–626 (1981).

[b2] Giovanetti V., Lloyd S. & Maccone L. Advances in quantum metrology. Nat. Photonics. 5, 222–229 (2011).

[b3] Lloyd S. Computational capacity of the universe. Phys. Rev. Lett. 88, 237901 (2002). 12059399 10.1103/PhysRevLett.88.237901

[b4] Caneva T., Murphy M., Calarco T., Fazio R., Montangero S., Giovannetti V. & Santoro G. E. Optimal control at the quantum speed limit. Phys. Rev. Lett. 103, 240501 (2009). 20366188 10.1103/PhysRevLett.103.240501

[b5] Mandelstam L. & Tamm I. The uncertainty relation between energy and time in nonrelativistic quantum mechanics. J. Phys. (USSR) 9, 249–254 (1945).

[b6] Fleming G. N. A unitarity bound on the evolution of nonstationary states. Nuovo Cimento A 16, 232–240 (1973).

[b7] Anandan J. & Aharonov Y. Geometry of quantum evolution. Phys. Rev. Lett. 65, 1697–1700 (1990). 10042340 10.1103/PhysRevLett.65.1697

[b8] Vaidman L. Minimum time for the evolution to an orthogonal quantum state. Am. J. Phys. 60, 182–183 (1992).

[b9] Margolus N. & Levitin L. B. The maximum speed of dynamical evolution. Phys. D 120, 188–195 (1998).

[b10] Levitin L. B. & Toffoli T. Fundamental limit on the rate of quantum dynamics: the unified bound is tight. Phys. Rev. Lett. 103, 160502 (2009). 19905679 10.1103/PhysRevLett.103.160502

[b11] Giovannetti V., Lloyd S. & Maccone L. Quantum limits to dynamical evolution. Phys. Rev. A 67, 052109 (2003).

[b12] Jones P. & Kok P. Geometric derivation of the quantum speed limit. Phys. Rev. A 82, 022107 (2010).

[b13] Deffner S. & Lutz E. Energy-time uncertainty relation for driven quantum systems. J. Phys. A: Math. Theor. 46, 335302 (2013).

[b14] Pfeifer P. How fast can a quantum state change with time? Phys. Rev. Lett. 70, 3365–3368 (1993). 10053850 10.1103/PhysRevLett.70.3365

[b15] Pfeifer P. & Fröhlich J. Generalized time-energy uncertainty relations and bounds on lifetimes of resonances. Rev. Mod. Phys. 67, 759–779 (1995).

[b16] Taddei M. M., Escher B. M., Davidovich L. & de Matos Filho R. L. Quantum speed limit for physical processes. Phys. Rev. Lett. 110, 050402 (2013). 23414007 10.1103/PhysRevLett.110.050402

[b17] del Campo A., Egusquiza I. L., Plenio M. B. & Huelga S. F. Quantum speed limits in open system dynamics. Phys. Rev. Lett. 110, 050403 (2013). 23414008 10.1103/PhysRevLett.110.050403

[b18] Deffner S. & Lutz E. Quantum speed limit for non-Markovian dynamics. Phys. Rev. Lett. 111, 010402 (2013). 23862985 10.1103/PhysRevLett.111.010402

[b19] Addis C., Brebner G., Haikka P. & Maniscalco S. Coherence trapping and information back-flow in dephasing qubits. arXiv:1311.0699 (2013).

[b20] Audenaert K. M. R. Comparisons between quantum state distinguishability measures. arXiv:1207.1197.

[b21] von Neumann J. Some matrix-inequalities and metrization of matric-space. Tomsk Univ. Rev. 1, 286–300 (1937).

[b22] Grigorieff R. D. A note on von Neumann's trace inequalitv. Mathematische Nachrichten 151, 327–328 (1991).

[b23] Chin A. W., Huelga S. F. & Plenio M. B. Quantum metrology in non-Markovian environments. Phys. Rev. Lett. 109, 233601 (2012). 23368199 10.1103/PhysRevLett.109.233601

[b24] Fanchini F. F., Karpat G., Castelano L. K. & Rossatto D. Z. Probing the degree of non-Markovianity for independent and common environments. Phys. Rev. A 88, 012105 (2013).

[b25] Bureuer H. P. & Petruccione F. The Theory of Open Quantum Systems (Oxford University Press, New York, 2002).

[b26] Leggett A. J., Chakravarty S., Dorsey A., Fisher M., Garg A. & Zwerger W. Dynamics of the dissipative two-state system. Rev. Mod. Phys. 59, 1–85 (1987).

[b27] Haikka P., Johnson T. H. & Maniscalco S. Non-Markovianity of local dephasing channels and time-invariant discord. Phys. Rev. A 87, 010103(R) (2013).

[b28] Peres A., Scudo P. F. & Terno D. R. Quantum entropy and special relativity. Phys. Rev. Lett. 88, 230402 (2002). 12059340 10.1103/PhysRevLett.88.230402

[b29] Gingrich R. M. & Adami C. Quantum entanglement of moving bodies. Phys. Rev. Lett. 89, 270402 (2002). 12513186 10.1103/PhysRevLett.89.270402

[b30] Alsing P. M., Fuentes-Schuller I., Mann R. B. & Tessier T. E. Entanglement of Dirac fields in noninertial frames. Phys. Rev. A 74, 032326 (2006).

[b31] Wang J. C., Deng J. F. & Jing J. L. Classical correlation and quantum discord sharing of Dirac fields in noninertial frames. Phys. Rev. A 81, 052120 (2010).

[b32] Lamata L., Martin-Delgado M. A. & Solano E. Relativity and lorentz invariance of entanglement distillability. Phys. Rev. Lett. 97, 250502 (2006). 17280335 10.1103/PhysRevLett.97.250502

[b33] Fuentes-Schuller I. & Mann R. B. Alice falls into a black hole: entanglement in noninertial frames. Phys. Rev. Lett. 95, 120404 (2005). 16197056 10.1103/PhysRevLett.95.120404

[b34] Alsing P. M. & Milburn G. J. Teleportation with a uniformly accelerated partner. Phys. Rev. Lett. 91, 180404 (2003). 14611272 10.1103/PhysRevLett.91.180404

[b35] Xu Z. Y., Luo S. L., Yang W. L., Liu C. & Zhu S. Q. Quantum speedup in memory environment. arXiv:1311.1593 (2013).

